# The Japanese Breast Cancer Society Clinical Practice Guidelines for systemic treatment of breast cancer, 2022 edition

**DOI:** 10.1007/s12282-023-01505-x

**Published:** 2023-10-07

**Authors:** Mitsuo Terada, Aki Ito, Yuichiro Kikawa, Kei Koizumi, Yoichi Naito, Tatsunori Shimoi, Mikiya Ishihara, Takashi Yamanaka, Yukinori Ozaki, Fumikata Hara, Rikiya Nakamura, Masaya Hattori, Minoru Miyashita, Naoto Kondo, Tetsuhiro Yoshinami, Masahiro Takada, Koji Matsumoto, Kazukata Narui, Shinsuke Sasada, Takayuki Iwamoto, Mitsuchika Hosoda, Yuko Takano, Takaaki Oba, Hitomi Sakai, Akari Murakami, Toru Higuchi, Junko Tsuchida, Yuko Tanabe, Tomoko Shigechi, Emi Tokuda, Michiko Harao, Shinichiro Kashiwagi, Junichi Mase, Junichiro Watanabe, Shigenori E. Nagai, Chikako Yamauchi, Yutaka Yamamoto, Hiroji Iwata, Shigehira Saji, Tatsuya Toyama

**Affiliations:** 1 Department of Breast Surgery, Nagoya City University Graduate School of Medical Sciences, Nagoya, Japan; 2 Department of Breast Surgery, Akita Red Cross Hospital, Akita, Japan; 3 Department of Breast Surgery, Kansai Medical University Hospital, Hirakata, Japan; 4 Division of Breast Surgery, Department of First Surgery, Hamamatsu University School of Medicine, Hamamatsu, Japan; 5 Department of General Internal Medicine, National Cancer Center Hospital East, Kashiwa, Japan; 6 Department of Medical Oncology, National Cancer Center Hospital, Tokyo, Japan; 7 Department of Medical Oncology, Mie University Hospital, Tsu, Japan; 8 Department of Breast Surgery and Oncology, Kanagawa Cancer Center, Yokohama, Japan; 9 Department of Breast Medical Oncology, The Cancer Institute Hospital of Japanese Foundation for Cancer Research, Tokyo, Japan; 10 Division of Breast Surgery, Chiba Cancer Center, Chiba, Japan; 11 Department of Breast Oncology, Aichi Cancer Center Hospital, Nagoya, Japan; 12 Department of Breast and Endocrine Surgical Oncology, Tohoku University Graduate School of Medicine, Sendai, Japan; 13 Division of Breast Surgery, Ichikawa Geka, Ogaki, Japan; 14 Department of Breast and Endocrine Surgery, Graduate School of Medicine, Osaka University, Osaka, Japan; 15 Department of Breast Surgery, Kyoto University Graduate School of Medicine, Kyoto, Japan; 16 Medical Oncology Division, Hyogo Cancer Center, Akashi, Japan; 17 Department of Breast and Thyroid Surgery, Yokohama City University Medical Center, Yokohama, Japan; 18 Department of Surgical Oncology, Research Institute for Radiation Biology and Medicine, Hiroshima University, Hiroshima, Japan; 19 Breast and Thyroid Surgery, Kawasaki Medical School Hospital, Kurashiki, Japan; 20 Department of Breast Surgery, Hokkaido University Hospital, Sapporo, Japan; 21 Department of Clinical Oncology and Chemotherapy, Nagoya University Hospital, Nagoya, Japan; 22 Division of Breast and Endocrine Surgery, Department of Surgery, Shinshu University School of Medicine, Nagano, Japan; 23 Advanced Cancer Translational Research Institute, Showa University, Tokyo, Japan; 24 Department of Breast Center, Ehime University Hospital, Toon, Japan; 25 Breast Unit, Japanese Red Cross Saitama Hospital, Saitama, Japan; 26 Division of Digestive and General Surgery, Niigata University Graduate School of Medical and Dental Sciences, Niigata, Japan; 27 Department of Medical Oncology, Toranomon Hospital, Tokyo, Japan; 28 Department of Surgery and Science, Graduate School of Medical Sciences, Kyushu University, Fukuoka, Japan; 29 Department of Medical Oncology, School of Medicine, Fukushima Medical University, Fukushima, Japan; 30 Department of Breast Oncology, Jichi Medical University, Shimotsuke, Japan; 31 Department of Breast Surgery, Osaka Metropolitan University Graduate School of Medicine, Osaka, Japan; 32 Department of Breast Surgery, Gifu Prefectural General Medical Center, Gifu, Japan; 33 Department of Breast Oncology, Graduate School of Medicine, Juntendo University, Tokyo, Japan; 34 Division of Breast Oncology, Saitama Cancer Center, Ina, Japan; 35 Department of Radiation Oncology, Shiga General Hospital, Moriyama, Japan; 36 Department of Breast and Endocrine Surgery, Kumamoto University Hospital, Kumamoto, Japan

**Keywords:** Breast cancer, Guidelines, Systemic treatment, JBCS

## Abstract

The Japanese Breast Cancer Society (JBCS) Clinical Practice Guidelines for systemic treatment of breast cancer were updated to the 2022 edition through a process started in 2018. The updated guidelines consist of 12 background questions (BQs), 33 clinical questions (CQs), and 20 future research questions (FRQs). Multiple outcomes including efficacy and safety were selected in each CQ, and then quantitative and qualitative systematic reviews were conducted to determine the strength of evidence and strength of recommendation, which was finally determined through a voting process among designated committee members. Here, we describe eight selected CQs as important updates from the previous guidelines, including novel practice-changing updates, and recommendations based on evidence that has emerged specifically from Japanese clinical trials.

**Supplementary Information:**

The online version contains supplementary material available at 10.1007/s12282-023-01505-x.

## Introduction

The Japanese Breast Cancer Society (JBCS) first published its Clinical Practice Guidelines in 2002 and continues to update these guidelines regularly to remain current. The current JBCS Clinical Practice Guidelines for breast cancer include systemic treatment [1], surgical treatment [2], radiation treatment [3], screening and diagnosis [4], and epidemiology and prevention. Here, we present the updated JBCS Clinical Practice Guidelines for systemic treatment 2022 edition, which have been developed since 2018 through minor online updates (2019, 2020 and 2021). These guidelines have a distinguishing characteristic wherein they cover globally acknowledged evidence and practices, and also refer to evidence developed in Japan and are adapted to the clinical context in Japan. In this article, we list all background questions (BQs), clinical questions (CQs) and future research questions (FRQs), with addition of details on important CQs derived from the POTENT [5], monarchE [6], RESPECT [7], KEYNOTE-522 [8], MONALEESA-7 [9], SELECT BC [10], DESTINY-Breast03 [11], and OlympiA [12] trials. The guidelines provide important updates and current recommendations to better support shared decision-making process in systemic therapy for early and metastatic breast cancer. 

## Background questions (BQs) and clinical questions (CQs)

BQs are positioned as established standard treatments for important issues, whereas CQs correspond to important ones in clinical practice that involve uncertainty in making decisions and are evaluated based on a certain level of evidence. For each CQ, multiple outcomes including efficacy and safety were selected, and then strength of evidence (SoE), strength of recommendation (SoR), and consensus rate were determined by quantitative and qualitative systematic reviews and voting at recommendation decision meetings of the Clinical Guidelines Committee. SoE was classified into four levels: “Strong”, “Moderate”, “Weak”, and “Very weak” for each outcome. SoR was similarly classified into four levels: “Strongly recommended to do; 1”, “Weakly recommended to do; 2”, “Weakly recommended not to do; 3”, and “Strongly recommended not to do; 4”. All BQs and statements are listed in Table 1, and all CQs and recommendations are shown for early breast cancer (EBC) in Table 2 and for metastatic breast cancer (MBC) in Table 3.

**Table 1:** Background questions

*Early breast cancer (EBC)*	
BQ.1	Is endocrine therapy (ET) effective for patients with HR-positive EBC?
Statement	ET for patients with HR-positive EBC is effective
BQ.2	Does tamoxifen increase the risk of developing endometrial cancer (uterus cancer)?
Statement	Tamoxifen has been shown to increase the risk of developing endometrial cancer (uterus cancer), primarily in postmenopausal women, but does not significantly increase the risk of mortality. Gynecological examination is recommended for patients with symptoms such as irregular genital bleeding
BQ.3	Is systemic adjuvant therapy based on histological type recommended for breast cancer diagnosed as a special type by pathologic classification?
Statement	It is reasonable to apply the same systemic therapy as that for invasive ductal carcinoma of the breast in patients with special types of breast cancer, but this therapy should also take into account the characteristics of the histological type (prognosis, subtype trends, and sensitivity to systemic therapy)
BQ.4	Is systemic therapy similar to that for breast cancer recommended for axillary lymph node metastases (adenocarcinoma) with an unknown primary site?
Statement	The standard treatment for axillary lymph node metastases with an unknown primary site (occult breast cancer) is histopathologic examination of the metastatic axillary lymph nodes, surgical resection or radiation therapy, and systemic therapy similar to that used for breast cancer with positive axillary lymph nodes
*Metastatic breast cancer (MBC)*	
BQ.5	What is the most useful method of ovarian function suppression (OFS) for premenopausal patients with HR-positive MBC?
Statement	The most useful method of ovarian function suppression is not clear, although luteinizing hormone-releasing hormone (LH-RH) agonists, bilateral oophorectomy, and irradiation have been utilized
	LH-RH agonists are generally used in clinical practice, but toxicity, cost, and treatment duration should be considered in selecting treatment
BQ.6	Is anthracycline-based chemotherapy recommended as first- or second-line chemotherapy for patients with HER2-negative MBC?
Statement	Anthracycline-based chemotherapy is standard chemotherapy for patients who have not previously received neoadjuvant/adjuvant therapy
BQ.7	Is taxane-based chemotherapy recommended as first- or second-line chemotherapy for patients with HER2-negative MBC?
Statement	Taxane-based chemotherapy is standard first- and second-line chemotherapy for patients with HER2-negative MBC
BQ.8	Are bone-modifying agents (BMAs) (denosumab, zoledronic acid) recommended for bone metastasis of breast cancer?
Statement	BMAs have been shown to reduce the risk of skeletal-related events (SREs) associated with bone metastasis; thus, use of BMAs in combination with systemic therapy is standard for patients with bone metastasis
	Denosumab has been shown to significantly reduce the risk of SREs compared to zoledronic acid
*Others*	
BQ.9	Are various vaccinations recommended before or during chemotherapy?
Statement	It is reasonable for patients receiving chemotherapy to receive influenza vaccine, pneumococcal vaccine, and COVID-19 vaccine prior to chemotherapy
BQ.10	Is drug intervention recommended for endocrine-induced hot flashes and arthralgia?
Statement	Hormone replacement therapy should not be administered for hot flashes caused by ET
	Further research is needed to determine the efficacy of interventions with drugs such as selective serotonin reuptake inhibitors (SSRIs)
	Nonsteroidal anti-inflammatory drugs (NSAIDs) and acetaminophen should be used to alleviate arthralgia. If arthralgia cannot be managed using these drugs, ET should be changed
BQ.11	Are BMAs (bisphosphonates, denosumab) recommended for prevention and treatment of osteoporosis in patients using aromatase inhibitors (AIs)?
Statement	When using AIs, bone density should be regularly evaluated and BMAs should be administered based on the risk of fracture
BQ.12	Are complementary and alternative therapies recommended for treatment of breast cancer?
Statement	Complementary and alternative therapies should not be used to control progression of breast cancer or to prolong survival
	Complementary and alternative therapies can be considered for relief of symptoms and anxiety associated with standard cancer treatment

**Table 2:** Clinical questions for EBC

		SoR	SoE	Consensus rate
CQ.1	Is ET recommended after breast-conserving therapy for patients with HR-positive non-invasive ductal carcinoma of the breast?			
Recommendation	Tamoxifen is weakly recommended regardless of menopausal status	2	Strong	90%
	AIs are weakly recommended in postmenopausal patients	2	Strong	83%
CQ.2	What adjuvant ET is recommended for premenopausal patients with HR-positive EBC?			
Recommendation	Tamoxifen alone is strongly recommended for patients with high-risk HR-positive EBC	1	Strong	100%
	A combination of a LH-RH agonist and tamoxifen is strongly recommended	1	Strong	98%
	A combination of a LH-RH agonist and an AI is strongly recommended	1	Moderate	85%
CQ.3	What adjuvant ET is recommended for postmenopausal patients with HR-positive EBC?			
Recommendation	AI is strongly recommended	1	Strong	100%
	Tamoxifen is weakly recommended	2	Strong	96%
CQ.4	Is additional ET recommended after 5 years of adjuvant ET for patients with invasive breast cancer?			
Recommendation	Additional 5-year administration of tamoxifen after 5 years of tamoxifen is recommended	1–2	Moderate	1: 43% 2: 57%
	Additional 2- to 5-year administration of an AI after 5 years of ET is weakly recommended	2	Strong	98%
CQ.5	Is concurrent use of S-1 with ET recommended as adjuvant therapy for patients with HR-positive, HER2-negative EBC?			
Recommendation	Concurrent use of S-1 with ET for 1 year is strongly recommended for patients with a high risk of recurrence	1	Moderate	72%
CQ.6	Is abemaciclib combined with ET recommended as adjuvant therapy for patients with HR-positive, HER2-negative EBC?			
Recommendation	Concurrent use of abemaciclib with ET for 2 years is strongly recommended for patients with a high risk of recurrence	1	Moderate	75%
CQ.7	Is sequential administration of anthracycline- and taxane-based chemotherapy recommended for patients with HER2-negative EBC?			
Recommendation	Sequential administration of anthracycline- and taxane-based chemotherapy is strongly recommended for high-risk HER2-negative EBC	1	Strong	92%
CQ.8	Is TC recommended for patients with HER2-negative EBC treated with chemotherapy?			
Recommendation	TC is weakly recommended	2	Moderate	92%
CQ.9	Is dose-dense chemotherapy recommended for patients with EBC treated with chemotherapy?			
Recommendation	Dose-dense chemotherapy is strongly recommended for high-risk EBC	1	Strong	72%
CQ.10	Is capecitabine recommended as adjuvant chemotherapy for patients with HER2-negative EBC who did not achieve a pathologic complete response (pCR) with neoadjvuant chemotherapy?			
Recommendation	Six to eight cycles of capecitabine is strongly recommended	1	Moderate	77%
CQ.11	Is it recommended to omit adjuvant chemotherapy for patients with HR-positive, HER2-negative EBC based on the results of a multigene assay?			
Recommendation	If the RS of Oncotype DX is 25 or less, it is strongly recommended to omit adjuvant chemotherapy for patients with negative lymph nodes	1	Strong	90%
CQ.12	Is addition of pertuzumab to trastuzumab recommended for patients with HER2-positive EBC treated with neoadjuvant chemotherapy?			
Recommendation	Addition of pertuzumab to trastuzumab is strongly recommended	1	Strong	82%
CQ.13	Is trastuzumab emtansine recommended as adjuvant therapy for patients with HER2-positive EBC who did not achieve pCR with neoadjuvant chemotherapy?			
Recommendation	Trastuzumab emtansine 14 cycles is strongly recommended	1	Moderate	87%
CQ.14	Is addition of pertuzumab to trastuzumab recommended for patients with HER2-positive EBC treated with adjuvant chemotherapy?			
Recommendation	Addition of pertuzumab to trastuzumab is strongly recommended for patients with high-risk HER2-positive breast cancer	1	Strong	89%
CQ.15	Is trastuzumab monotherapy recommended as adjuvant therapy for elderly patients with HER2-positive EBC?			
Recommendation	Trastuzumab monotherapy is weakly recommended for elderly patients who have difficulty receiving chemotherapy	2	Weak	98%
CQ.16	Is an immune checkpoint inhibitor recommended as neoadjuvant/adjuvant therapy for patients with triple-negative EBC?			
Recommendation	Pembrolizumab (an anti-PD-1 antibody) is weakly recommended	2	Moderate	80%
CQ.17	Is platinum-based chemotherapy recommended for patients with triple-negative EBC?			
Recommendation	Platinum-based chemotherapy is strongly recommended,	1	Strong	70%
CQ.33 (former FRQ.5)	Are polyADP-ribose polymerase (PARP) inhibitors recommended as adjuvant therapy for patients with germline *BRCA1/2* pathogenic variant-positive EBC?			
Recommendation	Olaparib for 1 year after perioperative chemotherapy is strongly recommended for patients with HER2-negative EBC and a high risk of recurrence	1	Moderate	90%

**Table 3:** Clinical questions for MBC

		SoR	SoE	Consensus rate
CQ.18	What is recommended as first-line ET for premenopausal patients with HR-positive, HER2-negative MBC?			
Recommendation	OFS in combination with a cyclin-dependent kinase (CDK)4/6 inhibitor and a nonsteroidal aromatase inhibitor (NSAI) is recommended	1–2	Weak	1: 53% 2: 47%
	The combination of OFS and ET alone is weakly recommended			
	The combination of OFS and tamoxifen is weakly recommended	2	Moderate	95%
	The combination of OFS and a NSAI is weakly recommended	2	Weak	100%
CQ.19	What is recommended as second-line or subsequent ET for premenopausal patients with HR-positive, HER2-negative MBC?			
Recommendation	Fulvestrant and a CDK4/6 inhibitor in combination with a LH-RH agonist is strongly recommended	1	Moderate	97%
	OFS in conjunction with an AI or other ET used for postmenopausal patients is weakly recommended	2	Weak	97%
CQ.20	What is recommended as ET for postmenopausal patients with HR-positive, HER2-negative MBC?			
Recommendation	The combination of a NSAI and a CDK4/6 inhibitor is strongly recommended	1	Strong	100%
	Fulvestrant alone is weakly recommended	2	Weak	97%
	An AI alone is weakly recommended	2	Moderate	91%
CQ.21	What is recommended as second-line ET when an AI is administered as first-line therapy for postmenopausal patients with HR-positive, HER2-negative MBC?			
Recommendation	The combination of fulvestrant and a CDK4/6 inhibitor is strongly recommended	1	Strong	100%
CQ.22	What is recommended as third-line or later ET for postmenopausal patients with HR-positive, HER2-negative MBC?			
Recommendation	The combination of exemestane and everolimus is weakly recommended for NSAI-refractory MBC	2	Weak	98%
CQ.23	Is bevacizumab in combination with chemotherapy recommended as first- or second-line chemotherapy for patients with HER2-negative MBC?			
Recommendation	Bevacizumab in combination with chemotherapy is weakly recommended	2	Strong	97%
CQ.24	Are oral fluoropyrimidines recommended as first- or second-line chemotherapy for patients with HER2-negative MBC?			
	First-line chemotherapy:			
Recommendation	S-1 is weakly recommended	2	Moderate	86%
	Capecitabine is weakly recommended	2	Weak	86%
	Second-line chemotherapy:			
Recommendation	S-1 or capecitabine is weakly recommended	2	Weak	100%
CQ.25	Is eribulin recommended as first- or second-line chemotherapy for patients with HER2-negative MBC?			
Recommendation	Eribulin is weakly recommended for patients previously treated with anthracycline- and taxane-based chemotherapy, including in neoadjuvant/adjuvant therapy	2	Weak	92%
CQ.26	Is trastuzumab + pertuzumab + taxane recommended as first-line therapy for patients with HER2-positive MBC?			
Recommendation	The combination of trastuzumab + pertuzumab + docetaxel is strongly recommended	1	Strong	100%
	The combination of trastuzumab + pertuzumab + paclitaxel is weakly recommended	2	Moderate	97%
CQ.27	Is trastuzumab emtansine recommended as first-line therapy for patients with HER2-positive MBC?			
Recommendation	Trastuzumab emtansine is weakly not recommended	3	Weak	79%
CQ.28	Is trastuzumab deruxtecan (T-DXd) recommended as second-line therapy for patients with HER2-positive MBC?			
Recommendation	T-DXd is strongly recommended as second-line therapy for patients with HER2-positive MBC that has progressed during or after the combination of trastuzumab, pertuzumab and chemotherapy	1	Moderate	90%
CQ.29	Is ET alone or in combination with anti-HER2 therapy recommended for patients with HER2-positive, HR-positive MBC?			
Recommendation	Anti-HER2 therapy in combination with ET is weakly recommended for patients with HER2-positive, HR-positive MBC that is unsuitable for chemotherapy	2	Moderate	88%
	ET alone is weakly not recommended for patients with HER2-positive, HR-positive MBC that is unsuitable for chemotherapy	3	Moderate	76%
CQ.30	Is platinum-based chemotherapy recommended for patients with triple-negative MBC?			
Recommendation	Platinum-based chemotherapy is weakly recommended	2	Weak	98%
CQ.31	Are PD-1/PD-L1 inhibitors recommended for patients with MBC?			
Recommendation	Atezolizumab in combination with nanoparticle albumin-bound paclitaxel is strongly recommended for patients with PD-L1-positive triple-negative breast cancer	1	Moderate	94%
	Pembrolizumab in combination with chemotherapy (nanoparticle albumin-bound paclitaxel, paclitaxel, carboplatin plus gemcitabine) is strongly recommended for patients with PD-L1-positive triple-negative breast cancer	1	Moderate	97%
CQ.32	Are PARP inhibitors recommended for MBC patients with germline *BRCA1/2* pathogenic variants?			
Recommendation	Monotherapy with a PARP inhibitor is strongly recommended for patients with anthracycline- and taxane-based chemotherapy	1	Strong	88%

## Important updates on recommendations

We highlight eight CQs as important topics that include novel practice-changing updates and recommendations based on clinical trials in Japanese patients:

CQ.5 Is concurrent use of S-1 with endocrine therapy (ET) recommended as adjuvant therapy for patients with hormone receptor (HR)-positive, human epidermal growth factor receptor 2 (HER2)-negative EBC?

CQ.6 Is abemaciclib combined with ET recommended as adjuvant therapy for patients with HR-positive, HER2-negative EBC?

CQ.15 Is trastuzumab monotherapy recommended as adjuvant therapy for elderly patients with HER2-positive EBC?

CQ.16 Is an immune checkpoint inhibitor recommended as neoadjuvant/adjuvant therapy for patients with triple-negative EBC?

CQ.18 What is recommended as first-line ET for premenopausal patients with HR-positive, HER2-negative MBC?

CQ.24 Are oral fluoropyrimidines recommended as first- or second-line chemotherapy for patients with HER2-negative MBC?

CQ.28 Is trastuzumab deruxtecan (T-DXd) recommended as second-line therapy for patients with HER2-positive MBC?

CQ.33 (former FRQ.5) Are polyADP-ribose polymerase (PARP) inhibitors recommended as adjuvant therapy for patients with germline *BRCA1/2* pathogenic variant-positive EBC?

### CQ.5 Is concurrent use of S-1 with ET recommended as adjuvant therapy for patients with HR-positive, HER2-negative EBC?

Recommendation: Concurrent use of S-1 with ET for 1 year is strongly recommended for patients with a high risk of recurrence [SoR: 1; SoE: Moderate; consensus rate: 72% (31/43)].

S-1 is a combination of tegafur, which is a 5-fluorouracil (FU) prodrug, and two modulators, gimeracil and oteracil potassium. In Japan, S-1 was previously reimbursed by insurance when used for advanced recurrent breast cancer. In 2022, the indication was expanded to adjuvant therapy for HR-positive HER2-negative breast cancer with a high risk of recurrence based on the results of the POTENT trial [5].

The POTENT trial was a multicenter cooperative non-blinded randomized Phase III study of the efficacy and safety of addition of S-1 to adjuvant ET [5]. Eligibility criteria were patients with estrogen receptor (ER)-positive and HER2-negative primary breast cancer of Stages I–IIIB with a moderate or high risk of recurrence. This risk was defined as patients treated with neoadjuvant chemotherapy who showed positive axillary lymph node metastasis before surgery or pathological residual disease after neoadjuvant chemotherapy, and those who underwent surgery first and were positive for axillary lymph node metastasis or negative for axillary lymph node metastasis with other risk factors, regardless of adjuvant chemotherapy [5]. In ET, tamoxifen or toremifene was allowed in concurrent use with ovarian function suppression (OFS) for premenopausal patients, while anastrozole, letrozole or exemestane was used for postmenopausal patients. Oral S-1 80–120 mg/day was administered twice a day for 14 days with 7 days off concurrently with ET for 1 year.

A total of 1930 patients were randomly divided into the S-1 group (*n* = 957) and the ET alone group (*n* = 973). In an interim analysis after a median follow-up period of 52.2 months, the primary end point, invasive disease-free survival (iDFS) showed additional effects of S-1 (HR: 0.63, 95% CI 0.49–0.81, *p* = 0.0003) compared to ET alone, with 5-year iDFS rates of 87% and 82% in the S-1 and ET alone groups, respectively. However, there was no clear difference in overall survival (OS) in the interim analysis, and thus results from long-term follow-up are required. Adverse events of grade 3 or higher occurred at a higher rate with S-1 than with ET alone, and included neutropenia (8%), diarrhea (2%), leukocytopenia (2%), elevated bilirubin (1%), and fatigue (< 1%).

We determined the SoE as “Moderate” despite the POTENT trial being a well-planned high-quality study because the efficacy of adjuvant S-1 was shown in only one clinical study. In the guidelines, concurrent use of S-1 with ET for 1 year is strongly recommended because the advantages of S-1 seem to be greater than the disadvantages based on the improvement of 5% in the 5-year iDFS rate in the intention-to-treat (ITT) group, although adverse events increased. The inclusion criteria based on the risk of recurrence should be determined with reference to the inclusion and exclusion criteria of the POTENT study. For patients eligible for both the POTENT and monarchE trials (refer to CQ.6), treatment regimens should be selected based on the advantages and disadvantages of both agents and the patient preference (Supplementary Table 1).

### CQ.6 Is abemaciclib combined with ET recommended as adjuvant therapy for patients with HR-positive, HER2-negative EBC?

Recommendation: Concurrent use of abemaciclib with ET for 2 years is strongly recommended for patients with a high risk of recurrence [SoR: 1; SoE: Moderate; consensus rate: 75% (27/36)].

Use of a cyclin-dependent kinase (CDK) 4/6 inhibitor as adjuvant therapy for pre- and postmenopausal HR-positive HER2-negative breast cancer was examined in the monarchE (abemaciclib) [6], PALLAS (palbociclib) [13], NATALEE (ribociclib/not approved in Japan) [14], and PENELOPE-B (palbociclib) [15] trials.

The monarchE study was a multicenter cooperative non-blind randomized Phase III trial in patients with HR-positive HER2-negative breast cancer with a high risk of recurrence that examined addition of 2-year concurrent administration of abemaciclib to standard adjuvant ET after standard treatments such as surgery, neoadjuvant/adjuvant chemotherapy, and radiotherapy [6]. Patients with 1) ≥ 4 axillary lymph node metastases or 2) 1–3 axillary lymph node metastases with a tumor diameter ≥ 5 cm or histological grade 3 were included in cohort 1; and those with 1–3 axillary lymph node metastases with a tumor diameter < 5 cm, histological grade 1 or 2, and Ki67 ≥ 20% were included in cohort 2. A total of 5,637 patients were registered (abemaciclib group: n = 2,808, ET alone group: *n* = 2829). Cohort 1 included 5,120 patients, and cohort 2, for which registration was commenced 1 year later than cohort 1, included 517 patients. In the ITT analysis (cohorts 1 and 2) at a median follow-up period of 27.1 months, the 3-year iDFS rates were 88.8% and 83.4% in the abemaciclib and ET alone groups, respectively (HR: 0.70, 95% CI 0.59–0.82, *p* < 0.0001), showing an absolute benefit of 5.4% [16]. There was no difference in iDFS events between the groups because the observation period for cohort 2 was short. The incidences of grade 3 or higher adverse events were 49.2% and 15.9% in the abemaciclib and ET alone groups, respectively, showing a higher incidence with abemaciclib. Side effects with a high incidence (all grades) included diarrhea (83.5% vs. 8.6%), neutropenia (45.8% vs. 5.6%), and fatigue (40.6% vs. 17.8%); and care is required for adverse events (all grades) of thrombosis (2.5% vs. 0.6%) and interstitial pneumonia (3.2% vs. 1.3%).

The PALLAS and PENELOPE-B trials evaluated addition of palbociclib to ET for Stage II-III patients and non-pCR patients after neoadjuvant chemotherapy, respectively, for HR+/HER2-EBC. Palbociclib + ET did not show improved iDFS. In the NATALEE trial, addition of ribociclib (which is not approved in Japan) to ET showed improvement of 3-year iDFS in patients with HR+/HER2-EBC with a well-tolerated safety profile [14].

Abemaciclib as adjuvant therapy was approved based on the inclusion criteria for cohort 1 of the monarchE study in Japan. Currently, the only available data are from a single trial, which is a well-designed randomized controlled trial, and the SoE was determined to be “Moderate”. The guideline committee concluded that the 5.4% improvement in 3-year iDFS was substantially important. Based on this, 2-year use of abemaciclib concurrent with ET was determined to be strongly recommended for patients with a high risk of recurrence, after discussing the level of evidence, balance of advantages and disadvantages. However, the efficacy of the drug requires further confirmation in long-term follow-up.

### CQ.15 Is trastuzumab monotherapy recommended as adjuvant therapy for elderly patients with HER2-positive EBC?

Recommendation**:** Trastuzumab monotherapy is weakly recommended for elderly patients who have difficulty receiving chemotherapy [SoR: 2; SoE: Weak; consensus rate: 98% (46/47)].

Four randomized Phase III trials of adjuvant anti-HER2 therapy for patients with HER2-positive EBC showed improved DFS and OS in a subgroup analysis of patients aged ≥ 60 years old, although the ratio of elderly patients was low [17–20]. Thus, concurrent use of chemotherapy and anti-HER2 therapy is recommended, even for elderly patients. However, some elderly patients have difficulty receiving concomitant chemotherapy due to adverse events and comorbidities.

The RESPECT trial was a randomized Phase III trial performed in Japan to examine the non-inferiority of trastuzumab monotherapy to trastuzumab plus chemotherapy as adjuvant therapy for elderly patients with HER2-positive EBC [7]. In the study, 275 HER2-positive breast cancer patients aged ≥ 70 and ≤ 80 years old with Stage I–III. A disease was divided into a trastuzumab group and a standard therapy (chemotherapy + trastuzumab) group. The 3-year DFS rates (the primary end point) were 89.5% and 93.8% in the trastuzumab and chemotherapy + trastuzumab groups, respectively (HR: 1.36, 95% CI 0.72–2.58, *p* = 0.51), which was higher than the preset upper limit of non-inferiority (95% CI 1.69); thus, the non-inferiority of trastuzumab monotherapy was not demonstrated.

In an additional complementary analysis using the restricted mean survival time (RMST), the difference in RMST for DFS between the study arms at 3 years was − 0.39 months (95% CI − 1.71 to 0.93, *p* = 0.56). The 3-year OS was 97.2% vs. 96.6% in the trastuzumab vs. chemotherapy + trastuzumab groups (HR: 1.07, 95% CI 0.36–3.19). Adverse events included anorexia (7.4% vs. 44.3%, *p* < 0.0001) and hair loss (2.2% vs. 71.7%, *p* < 0.0001). Grade 3–4 non-hematological adverse events (11.9% vs. 29.8%, *p* = 0.0003) were more than twice as frequent in the chemotherapy + trastuzumab group, and grade 4 hematological adverse events were also significantly higher in this group (0% vs. 13.7%, *p* < 0.0001). Health-related QOL in the trastuzumab group was significantly better maintained for 1 year after commencement of the study [21].

Since only one randomized trial is available for this CQ, the SoE was determined as “Weak.” The intent is that the trial was a negative study because non-inferiority could not be statistically proven, but the absolute difference is small, so the clinical interpretation is that trastuzumab monotherapy is acceptable. This suggests that trastuzumab monotherapy is an option for elderly patients who cannot tolerate chemotherapy. It is weakly recommended to select patients for this therapy with reference to the inclusion criteria of the RESPECT trial.

### *CQ.16* Is an immune checkpoint inhibitor recommended as neoadjuvant/adjuvant therapy for patients with triple-negative EBC?

Recommendation**:** Pembrolizumab (anti-PD-1 antibody) is weakly recommended [SoR: 2; SoE: Moderate; consensus rate: 80% (32/40)].

The usefulness of a concurrent immune checkpoint inhibitor with perioperative chemotherapy for triple-negative breast cancer has been examined in two randomized Phase III studies [8, 22, 23]. The inclusion criteria in these studies required that the patients had a tumor diameter of T2 or larger or T1c with lymph node metastasis. In both studies, immune checkpoint inhibitors were used pre- and postoperatively. The KEYNOTE-522 trial evaluated neoadjuvant pembrolizumab (an anti-PD-1 antibody) plus chemotherapy, followed by adjuvant pembrolizumab in patients with triple-negative EBC. This trial showed pCR rates of 64.8% and 51.2% (*p* < 0.001), and estimated event-free survival (EFS) at 36 months of 84.5% and 76.8% (HR: 0.63, 95% CI 0.48–0.82, *p* < 0.001) in the pembrolizumab and placebo groups, respectively, indicating a significant improvement with pembrolizumab. The IMpassion031 trial evaluated neoadjuvant atezolizumab (an anti-PD-L1 antibody) plus chemotherapy, followed by adjuvant atezolizumab in patients with triple-negative EBC. In the ITT population, this trial showed pCR rates of 58% and 41% in the atezolizumab and placebo groups, respectively (*p* = 0.0044), indicating favorable results with atezolizumab. EFS (HR 0.76 (95% CI 0.4–1.44) was also reported, but the survival data remain immature.

Adverse events of grade 3 or higher occurred at rates of 76.8% and 72.2% in the pembrolizumab and placebo groups, respectively, in KEYNOTE-522, and at 57% and 53% in the atezolizumab and placebo groups, respectively, in IMpassion031. In both studies, there were increases in immune-related adverse events (irAEs), and certain irAEs may be irreversible and require long-term therapy.

Since each of the two drugs related to this CQ was examined in one randomized trial, we determined the SoE as “Moderate.” Concurrent use of pembrolizumab as perioperative chemotherapy for triple-negative breast cancer improved EFS and pCR, and we judged that the advantages are greater than the disadvantages, although serious adverse events and irAEs may be increased by pembrolizumab. With atezolizumab, the pCR rate improved, but data on prognosis are immature. In the recommendation decision meeting, voting was performed only for concurrent use of pembrolizumab, and pembrolizumab is weakly recommended based on the consensus rate.

### CQ.18 What is recommended as first-line ET for premenopausal patients with HR-positive, HER2-negative MBC?

Recommendation: OFS in combination with a CDK4/6 inhibitor and a nonsteroidal aromatase inhibitor (NSAI) is recommended [SoR: 1–2 (no agreement reached*); SoE: Weak; consensus rate: strong recommendation: 53% (18/34), moderate recommendation: 47% (16/34)]. The SoR was rated 1–2 since a consensus of > 70% was not reached even after three rounds of voting. The combination of OFS and ET alone is weakly recommended. The combination of OFS and tamoxifen is weakly recommended [SoR: 2; SoE: Moderate; consensus rate: 95% (39/41)]. The combination of OFS and a NSAI is weakly recommended [SoR: 2; SoE: Weak; consensus rate: 100% (41/41)].

ET with or without molecular targeted drugs should be considered for premenopausal HR-positive HER2-negative MBC as first-line treatment, if not in a life-threatening situation. In this CQ, we define first-line ET as the initial treatment for MBC irrespective of the timing of recurrence.

The efficacy of a CDK4/6 inhibitor in first-line ET for premenopausal HR-positive HER2-negative MBC has only been examined in one study (MONALEESA-7), in which addition of ribociclib, a CDK4/6 inhibitor, was examined in concurrent use of tamoxifen, letrozole or anastrozole with goserelin. The results suggested that ribociclib addition significantly improved progression-free survival (PFS) compared to placebo (23.8 vs. 13.0 months, HR: 0.55, 95% CI 0.44–0.69, *p* < 0.0001) [24] and improved OS (not reached vs. 40.9 months, HR: 0.71, 95% CI 0.54–0.95, *p* < 0.00973) [25]. Ribociclib has a side effect of QTc prolongation, and this side effect had the highest incidence with concurrent tamoxifen. This study showed the efficacy of ribociclib, including prolonged survival, which indicates that concurrent use of a CDK4/6 inhibitor is desirable for this patient population.

In Japan, palbociclib and abemaciclib are approved as CDK4/6 inhibitors, but ribociclib is not. There are no pivotal studies with results of these drugs in premenopausal patients, and thus, no clear evidence is available. However, the efficacy of three CDK4/6 inhibitors, palbociclib, abemaciclib and ribociclib, for improvement of PFS in HR-positive HER2-negative metastatic/recurrent breast cancer has been demonstrated in all Phase III clinical studies of postmenopausal first-line ET and pre-/postmenopausal second-line ET [9, 26–35]. Although no clinical data are available for drug and pre-/postmenopausal differences, failure to use CDK4/6 inhibitors due to a lack of clinical trial data is likely to cause a significant disadvantage to patients. Thus, the guidelines recommend ET with concurrent use of a CDK4/6 inhibitor as first-line therapy for premenopausal patients. For this therapy, use of a NSAI + OFS is recommended based on the efficacy and safety in the MONALEESA-7 trial. However, attention should be paid to increased adverse events and the financial burden associated with concurrent use of a CDK4/6 inhibitor.

### CQ.24 Are oral fluoropyrimidines recommended as first- or second-line chemotherapy for patients with HER2-negative MBC?

For first-line therapy, S-1 is weakly recommended [SoR: 2; SoE: Moderate; consensus rate: 86% (38/44)] and capecitabine is weakly recommended [SoR: 2; SoE: weak; consensus rate: 86% (38/44)]. Oral fluoropyrimidines have an advantage of causing no hair loss. Recommendation of these drugs as first-line chemotherapy was examined through comparison with other drugs.

The SELECT BC and SELECT BC-CONFIRM trials evaluated the non-inferiority of S-1 as first-line treatment relative to taxane and anthracycline, respectively [10, 36]. In both studies, taxane was used for about 30% of the subjects perioperatively. For OS, the hazard ratios of S-1 were 1.05 (95% CI 0.86–1.27) relative to taxane in the SELECT BC trial, and 1.09 (95% CI 0.80–1.48) relative to anthracycline in the SELECT BC-CONFIRM trial. In a scheduled integration analysis of these studies, the hazard ratio of S-1 relative to standard treatment (taxane + anthracycline) was 1.06 (95% CI 0.90–1.25). PFS tended to be favorable with the standard treatment (HR: 1.16, 95% CI 1.00–1.35). In QOL evaluation, S-1 was superior to taxane and equivalent to anthracycline. These drugs have different toxicological profiles: anthracycline and taxane have a high incidence of hair loss and peripheral neuropathy, and S-1 has a high incidence of diarrhea.

Capecitabine monotherapy as first-line therapy has been examined in four studies, two of which compared it with doxorubicin hydrochloride pegylated liposomes and 2 with CMF (cyclophosphamide, methotrexate, 5-FU) [37–40]. Patients administered anthracycline and taxane perioperatively accounted for 10–30% of cases in these studies. In one trial comparing capecitabine with doxorubicin hydrochloride pegylated liposomes, which included many elderly patients, there were no differences in OS, PFS, and overall response rate (ORR). An integration analysis of the two trials with CMF showed no difference in PFS and ORR, but capecitabine was superior for OS (HR: 0.71, 95% CI 0.56–0.91). However, it should be noted that CMF was the control. Among eight8 trials in which combination therapy with capecitabine and another drug was compared to combination therapy with anthracycline and taxane, those that could be included in a meta-analysis showed no differences for OS, PFS and ORR.

The SoEs of these drugs were determined by taking into consideration the bias risks of individual trials. For studies in which capecitabine monotherapy was used as the intervention, regimens that are currently not recognized as standard treatments were used as controls, and thus, the SoE was determined to be “Weak.” Regarding the balance of advantages and disadvantages, OS in subjects with S-1 was generally not lower than that with anthracycline or taxane, while S-1 was superior to taxane for higher QOL without hair loss. Therefore, we concluded that the advantages of S-1 outweighed the disadvantages as first-line chemotherapy. Data for comparison of capecitabine monotherapy with anthracycline and taxane as first-line treatment are insufficient, but superiority to CMF was confirmed without hair loss. Thus, we concluded that capecitabine has more advantages than disadvantages as first-line chemotherapy.

### CQ.28 Is T-DXd recommended as second-line therapy for patients with HER2-positive MBC?

Recommendation: T-DXd is strongly recommended as second-line therapy for patients with HER2-positive MBC that has progressed during or after the combination of trastuzumab, pertuzumab and chemotherapy [SoR: 1; SoE: Moderate; consensus rate: 90% (36/40)].

The safety and efficacy of T-DXd in patients with HER2-positive MBC that progressed during or after administration of trastuzumab + taxane were examined in the DESTINY-Breast03 trial [11]. This was a non-blind randomized Phase III trial comparing T-DXd and T-DM1 in 524 subjects, including patients with HER2-positive MBC with aggravation after treatment including taxane + trastuzumab, or with recurrent breast cancer within 6 months after pre- or postoperative administration of drugs including taxane + trastuzumab. As the primary endpoint, median PFS was not reached in the T-DXd group and was 6.8 months (HR: 0.28, 95% CI 0.22–0.37) in the T-DM1 group, showing significant improvement in the T-DXd group. The median OS was not reached in both groups in the second interim analysis, but there was significant improvement in the T-DXd group (HR: 0.64, 95% CI 0.47–0.87) [41]. Survival at 24 months after treatment was 77.4% (95% CI 71.7–82.1) and 69.9% (95% CI 63.7–75.2) in the T-DXd and T-DM1 groups, respectively.

Regarding safety, more cases developed interstitial pulmonary disease in the T-DXd group than in the T-DM1 group (27 [10.5%] vs. 5 [1.9%]). Adverse events of grade 3 or higher (T-DXd vs. T-DM1) included neutropenia (19.1% vs. 3.1%), leucopenia (6.6% vs. 0.4%), nausea (6.6% vs. 0.4%), vomiting (1.6% vs. 0.4%), and fatigue (5.1% vs. 0.8%). The only available data is from a single trial, which was a well-designed non-blind randomized controlled trial. A review of the study led to determination of the SoE as "Moderate." It is clear that T-DXd greatly improves the prognosis as second-line therapy. The drug does cause toxicity of interstitial pulmonary disease, but its advantages are greater than its disadvantages.

### CQ.33 (former FRQ5) Are PARP inhibitors recommended as adjuvant therapy for patients with germline BRCA1/2 pathogenic variant-positive EBC?

Recommendation: Olaparib for 1 year after perioperative chemotherapy is strongly recommended for patients with HER2-negative disease and a high risk of recurrence [SoR: 1; SoE: Moderate; consensus rate: 90% (66/73)]. We note that CQ.33 was elevated from a FRQ after publication of the 2022 edition of the guidelines. Therefore, the recommendation decision meeting was conducted on a different day to that for the other CQs, and a vote was held in April 2023.

A Phase III trial (OlympiA) showed the usefulness of postoperative addition of olaparib, a PARP inhibitor, in patients with germline *BRCA1/2* pathogenic variant-positive breast cancer with a high risk of recurrence who completed neoadjuvant/adjuvant chemotherapy [12, 42]. Comparison of administration of olaparib or a placebo for one year was performed in patients who underwent preoperative chemotherapy and had triple-negative breast cancer with non-pCR or HR-positive breast cancer with non-pCR and a CPS-EG score ≥ 3; and patients who underwent adjuvant chemotherapy and had triple-negative breast cancer of pT2 or higher or pN1 or higher or HR-positive breast cancer with ≥ 4 axillary lymph node metastases. At a median follow-up of 3.5 years, the 4-year OS rates were 89.8% and 86.4% in the olaparib and placebo groups, respectively (HR 0.68, 98.5% CI 0.47–0.97, *p* = 0.009) [42], and the 4-year iDFS rates were 82.7% and 75.4%, respectively (HR: 0.63, 95% CI 0.50–0.78), indicating significant improvements in OS and iDFS in the olaparib group.

Grade 3 or higher adverse events were observed at rates of 24.5% in the olaparib group and 11.3% in the placebo group (RR: 2.17, 95% CI 1.75–2.69). Grade ≥ 3 anemia occurred in 8.7% of patients in the olaparib group, but in only 0.3% in the placebo group. During a median follow-up of 3.5 years, the frequency of developing myelodysplastic syndrome/acute myeloid leukemia did not differ significantly between the groups (0.2% olaparib vs. 0.3% placebo, RR: 0.66, 95% CI 0.11–3.95).

The only available data are from a single trial, which was a well-designed randomized controlled trial in a specific patient population with high-risk breast cancer carrying germline *BRCA1/2* pathogenic variants. After a review of this study, we determined the SoE to be "Moderate." Despite the increased incidence of anemia and grade 3 or higher adverse events associated with olaparib, the significant improvements in OS and iDFS led to the conclusion that olaparib has more advantages than disadvantages.

## Future research questions (FRQs)

As described above, there have been remarkable advances in chemotherapy and targeted therapy for breast cancer. However, there are still unresolved issues. In these guidelines, clinical questions for which there are still insufficient data, but which are important issues, are listed as FRQs in Supplementary Table 2. The FRQs include consideration of comprehensive cancer genome profiling (CGP) tests, ET after CDK4/6 inhibitor therapy, adjuvant therapy for small tumors without lymph node metastasis, and systemic therapy for elderly patients.

## Conclusion

The JBCS Clinical Practice Guidelines for systemic treatment of breast cancer, 2022 edition includes significant updates from the 2018 edition. The complete version of these guidelines, which is available only in Japanese, can be accessed on the JBCS official website [43]. In clinical settings, it is essential to make decisions based on current evidence, as well as the patient's desires and social background. The JBCS Clinical Practice Guidelines provide recommendations that are adapted for the Japanese clinical environment and serve as practical tools for shared decision-making with patients and their families in the systemic treatment of breast cancer.

### Supplementary Information

Below is the link to the electronic supplementary material.

Supplementary file1 (PDF 197 kb)

## Data Availability

Data sharing is not applicable to this article as no datasets were generated or analyzed during the current study.
