# Supplementary material for: The Japanese Breast Cancer Society Clinical Practice Guidelines for systemic treatment of breast cancer, 2022 edition
Source: Breast Cancer. 2023 Oct 7;30(6):872–84. doi: 10.1007/s12282-023-01505-x (PMC10587293; doi:10.1007/s12282-023-01505-x)
Supplement: Supplementary file 1 — Supplementary file1 (PDF 197 kb) [file 12282_2023_1505_MOESM1_ESM.pdf]

Supplementary Table 1. Comparison of key results in the POTENT and monarchE trials

Item	POTENT	monarchE*
Number of patients	1,930 (S-1 + ET; 957 vs. ET; 973)	5,637 (Abemaciclib + ET; 2,808 vs. ET; 2,829)
Relative recurrence risk**	Moderate to high risk	High risk
Intervention	S-1 (twice daily) 2 weeks on / 1 week off<br>Duration: 1 year	Abemaciclib-continuous (twice daily)<br>Duration: 2 years
Discontinuation rate of intervention	198 (21%)	982 (17%)
Median follow-up time	55.2 months	27.1 months
Positivity of axillary lymph nodes	1,228 (63%)	5,622 (99%)
Number of positive nodes		
1 to 3	657 (34%)***	2,260 (40%)
≥4	189 (10%)***	3,362 (60%)
Neoadjuvant chemotherapy	392 (20%)	2,056 (36%)
Neoadjuvant or adjuvant chemotherapy	1,076 (56%)	5,376 (95%)
iDFS	HR 0.63 (95%CI 0.49-0.81)	HR 0.70 (95%CI 0.59-0.82)
DDFS/DRFS	101 (11%) vs 155 (16%)<br>(HR of DDFS not reported)	191 (6.8%) vs 278 (10%)<br>HR 0.69 (95%CI 0.57-0.83)
iDFS in patients with positive lymph nodes	HR 0.70 (95%CI 0.52-0.93)	HR 0.70 (95%CI 0.59-0.82)****

*The results of the monarchE study include data from Cohort 2, which is not subject to regulatory approval in Japan (at the time of publication of the 2022 edition of the guidelines).

** The monarchE trial included more high-risk patients than the POTENT trial.

*** The POTENT trial included 382 patients (20%) with unknown or missing information for lymph node metastases.

**** Including 15 patients (0.3%) with negative or missing data.

ET, endocrine therapy; iDFS, invasive disease-free survival; DDFS, distant disease-free survival; DRFS, distant relapse-free survival; HR, hazard ratio

Supplementary Table 2. Future Research Questions

EBC

FRQ.1	Is neoadjuvant ET recommended for patients with HR-positive HER2-negative invasive breast cancer?
Statement	The optimal duration of neoadjuvant ET and its impact on prognosis are unclear.<br>In postmenopausal women, treatment with AIs for at least 3 months is expected to improve breast conservation rates.<br>For premenopausal women, there is a lack of evidence for neoadjuvant ET for breast conservation and this is not recommended.<br>The efficacy of neoadjuvant ET is a prognostic factor, but postoperative treatment based on the response to neoadjuvant ET has yet to be established.
FRQ.2	Is omission of adjuvant ET recommended for patients with HR-positive breast cancer with an invasive diameter of 0.5 cm or less and negative lymph nodes?
Statement	Patients in whom adjuvant ET can be omitted are not clear from the clinicopathologic diagnosis.
FRQ.3	Is combination therapy with taxane and trastuzumab without anthracycline recommended as adjuvant therapy for patients with HER2-positive EBC?
Statement	Adjuvant therapy without anthracycline for patients with HER2-positive EBC may reduce adverse events such as heart failure without worsening the prognosis, but appropriate selection of drugs and target patients are issues for future studies.
FRQ.4	Is adjuvant chemotherapy recommended for triple-negative breast cancer with an invasive diameter of 1 cm or less and negative lymph nodes?
Statement	There are few data showing that adjuvant chemotherapy improves the prognosis for patients with triple-negative breast cancer with an invasive diameter of 1 cm or less and negative lymph nodes.<br>Predictors of response to chemotherapy and appropriate regimens for this group remain to be investigated.
FRQ.5	See important updates on recommendations "New CQ33"
FRQ.6	What is the recommended systemic therapy for early-stage male breast cancer?
	For ET, consider tamoxifen alone. If tamoxifen is difficult to use, an AI plus an LH-RH agonist may be considered.<br>Chemotherapy should be considered in accordance with early-stage female breast cancer.<br>Molecular-targeted therapy may be considered in accordance with early-stage female breast cancer.
FRQ.7	Is perioperative therapy recommended for early-stage elderly patients with breast cancer?
FRQ.7a	ET
Statement	AIs or tamoxifen are reasonable as adjuvant ET for patients with HR-positive breast cancer.
FRQ.7b	Chemotherapy
Statement	Standard chemotherapy is considered appropriate as perioperative chemotherapy for elderly patients with breast cancer.
FRQ.7c	Anti-HER2 therapy
Statement	In adjuvant chemotherapy for patients with HER2-positive breast cancer, it is reasonable to combine chemotherapy with anti-HER2 therapy.
FRQ.8	Is perioperative systemic therapy recommended for patients with EBC during pregnancy?
Statement	ET and molecular-targeted therapy, including anti-HER2 therapy, should not be administered during pregnancy.<br>Chemotherapy should not be given in the first trimester of pregnancy (0-14 weeks).<br>Chemotherapy in the second trimester (14-28 weeks) and third trimester (28 weeks or more) of pregnancy may be considered if deemed necessary,

	but long-term safety has not been established.
FRQ.9	Are BMAs recommended as adjuvant therapy to prevent recurrence in patients with EBC?
Statement	Administration of BMAs to prevent recurrence may reduce the risk of recurrence. However, the appropriate target, drug, dose, and duration of administration have yet to be established. A further problem is that there are no BMAs covered by Japanese insurance for patients with EBC.

MBC

FRQ.10	What is recommended as second-line ET for postmenopausal patients with HR-positive HER2-negative MBC? (If an AI alone is given as first-line ET, see CQ21)
FRQ.10a	When an AI and CDK4/6 inhibitor are used as first-line ET
Statement	The optimal second-line ET has not been established. Unused ETs (including combination therapy with molecular-targeted therapies such as mTOR inhibitors) may be considered.<br>Unused ETs (including combination therapy with molecular-targeted therapies such as mTOR inhibitors) may be considered.<br>There are no data that support readministration of CDK 4/6 inhibitors. Clinical trials are underway to address the mechanism of tolerance.
FRQ.10b	Monotherapy with fulvestrant as first-line ET
Statement	The optimal second-line ET has not been established.<br>Unused ETs (including concomitant use of molecular-targeted therapies such as CDK 4/6 inhibitors) may be considered.
FRQ.11	Is a PI3K inhibitor useful for patients with *PIK3CA* mutation-positive HR-positive HER2-negative MBC?
Statement	Efficacy of alpelisib (not approved in Japan) in patients with *PIK3CA* mutation-positive HR-positive HER2-negative MBC has been reported.
FRQ.12	What is the recommended treatment for patients with HER2-positive MBC in third-line and subsequent treatments?
Statement	If T-DXd is not used in second-line therapy, the usefulness of T-DXd in third-line therapy has been demonstrated.<br>When T-DXd is used for second-line therapy, drugs not used before the previous treatment should be considered for third-line and subsequent treatments, depending on the circumstances of each individual case, but there is insufficient evidence on the treatment sequence.<br>Several novel molecular-targeted agents have been approved by the FDA as third-line and subsequent therapies, but these agents are not approved in Japan.
FRQ.13	Is platinum therapy recommended for patients with MBC with germline *BRCA1/2* pathogenic variants?
Statement	The efficacy of platinum agents in MBC with germline *BRCA1/2* pathogenic variants is promising, but no randomized controlled trials have been performed for such cases.
FRQ.14	Is curative treatment recommended for patients with MBC?
Statement	There are reports of cases of MBC with a complete response to systemic therapy or in which additional local treatment (surgery, radiotherapy, etc.) resulted in undetectable tumor residuals on imaging, followed by long-term survival without relapse.<br>At present, the definition of the term "cure" and endpoints have not been established, and the validity of a treatment strategy targeting cure cannot be evaluated. Further studies need to be conducted.
FRQ.15	Is maintenance ET recommended after response to chemotherapy for patients with MBC?
Statement	Maintenance ET after chemotherapy response is likely to improve QOL compared to continued chemotherapy, but it is unclear whether the therapeutic effect can be maintained.
FRQ.16	What is the recommended systemic therapy for elderly patients with MBC?

Statement	AIs and fulvestrant for elderly patients with HR-positive MBC are likely to be as effective as in younger postmenopausal patients.<br>For combined ET and molecular-targeted therapy, progression free survival (PFS) is similar to that in younger patients, but toxicity may increase.<br>Chemotherapy (plus anti-HER2 therapy, bevacizumab, PD-1/PD-L1 inhibitors) for elderly patients with MBC may be as effective as in younger patients, but increased toxicity is a concern. Development and clinical application of appropriate geriatric assessments in Japanese patients are needed.
FRQ.17	What is the recommended systemic therapy for male patients with MBC?
Statement	For ET, consider tamoxifen alone, an AI plus LH-RH agonist, or fulvestrant alone.<br>There are no data on the superiority or inferiority of ETs and the order in which they should be administered.<br>Consider combining ET with a CDK4/6 inhibitor.<br>Chemotherapy and other endocrine and molecular-targeted therapies should be considered as for female patients with MBC.
FRQ.18	Is systemic therapy recommended after resection for local/regional recurrence?
Statement	After resection for local/regional recurrence, ET or chemotherapy should be considered based on the evidence for adjuvant systemic therapy and the patient's treatment history.
FRQ.19	Is systemic therapy recommended for brain metastases and meningeal dissemination?
Statement	Radiation therapy and surgery are treatment options for symptomatic brain metastases.<br>For patients with brain metastases that are controlled or asymptomatic after local therapy, systemic therapy is the treatment of choice.<br>There is no established treatment for meningeal dissemination of breast cancer.

Others

FRQ.20	Is comprehensive genomic profiling (CGP) using next-generation sequencing useful in breast cancer treatment?
Statement	The appropriate timing of CGP for MBC and its prognostic value are currently unclear, but this approach is being incorporated into drug development and clinical trials, and it is hoped that its usefulness will be clarified.<br>Japanese insurance covers CGP after completion of "standard treatment," which is generally defined as the "strongly recommended" treatment in each section of the guidelines. However, depending on each patient's situation, not all may be candidates for CGP.<br>Given that the time between submission of tumor tissue or blood and the return of results is 4 to 8 weeks, a CGP test should be performed during standard treatment to obtain results at the time of its expected completion.<br>Under the current situation in Japan (insurance coverage after completion of standard treatment), the possibility of participating in a clinical trial after a CGP test should also be considered (patient's general status, metastatic organs, etc.).<br>Access to facilities that provide clinical trials for molecular-targeted therapy should also be considered in the decision to use CGP, but this option should not be excluded simply because of the remoteness of the location. Currently, the problem is the small number of facilities providing clinical trials for molecular-targeted therapy in Japan.<br>In the future, it is likely that treatment development will be based on multiple CGP tests using liquid biopsy.
FRQ.21	Is systemic therapy recommended for patients with distant metastases of malignant phyllodes tumor?
Statement	There are no prospective trials that have examined the benefit of systemic therapy for malignant phyllodes tumor. Consider treatment as for soft-tissue sarcomas, with doxorubicin alone for first-line therapy and pazopanib, eribulin, or trabectedin for second-line or subsequent therapy.
